# Supplementary material for: Wastewater surveillance as a predictive tool for COVID-19: A case study in Chengdu
Source: PLoS One. 2025 May 28;20(5):e0324521. doi: 10.1371/journal.pone.0324521 (PMC12118905; doi:10.1371/journal.pone.0324521)
Supplement: S3 Table — (DOCX) [file pone.0324521.s003.docx]

Table S3. The results in different models.

| Models | Infected cases | | Infection rates | | Spearman's r |
| --- | --- | --- | --- | --- | --- |
|  | Min | Max | Min | Max |  |
| Model 1 | 6,517 | 486,384 | 0.01 | 3.27 | 0.898 |
| Model 2 | 6,781 | 506,095 | 0.01 | 3.41 | 0.897 |
| Model 3 | 9,204 | 686,982 | 0.02 | 4.62 | 0.899 |
| Model 4 | 6,517 | 486,384 | 0.01 | 2.95 | 0.897 |
| Model 5 | 6,517 | 486,384 | 0.01 | 3.6 | 0.901 |

Model 1: Persons Infected = $\frac{Cr*Q}{Cf\text{*211}+(Cru)*1.5}$; Population size = $\frac{CN*Q}{\text{4.2g}/person\cdot\text{day}}$;

Model 2: Persons Infected = $\frac{Cr*Q}{Cf\text{*211*82.4\%}+(Cru)*1.5}$; Population size = $\frac{CN*Q}{\text{4.2g}/person\cdot\text{day}}$;

Model 3: Persons Infected = $\frac{Cr*Q}{Cf\text{*128}+(Cru)*1.5}$; Population size = $\frac{CN*Q}{\text{4.2g}/person\cdot\text{day}}$;

Model 4: Persons Infected = $\frac{Cr*Q}{Cf\text{*211}+(Cru)*1.5}$; Population size = $\frac{CN*Q}{\text{4.2g*90\%}/person\cdot\text{day}}$;

Model 5: Persons Infected = $\frac{Cr*Q}{Cf\text{*211}+(Cru)*1.5}$; Population size = $\frac{CN*Q}{\text{4.2g*110\%}/person\cdot\text{day}}$;
