# Supplementary material for: Wastewater surveillance as a predictive tool for COVID-19: A case study in Chengdu
Source: PLoS One. 2025 May 28;20(5):e0324521. doi: 10.1371/journal.pone.0324521 (PMC12118905; doi:10.1371/journal.pone.0324521)
Supplement: Attachment — Submitted filename: Response to Reviewers.docx [file pone.0324521.s005.docx]

Dear Reviewers,

We greatly appreciate your comments and suggestions. In response to the reviewers' feedback, we have addressed each point raised by the academic editor and the reviewers. Additionally, we have made some changes to the manuscript, which are highlighted for your convenience.

**Authors Responses to Journal Requirements**

When submitting your revision, we need you to address these additional requirements.

1. Please ensure that your manuscript meets PLOS ONE's style requirements, including those for file naming. The PLOS ONE style templates can be found at

https://journals.plos.org/plosone/s/file?id=wjVg/PLOSOne_formatting_sample_main_body.pdf and

<https://journals.plos.org/plosone/s/file?id=ba62/PLOSOne_formatting_sample_title_authors_affiliations.pdf>

**AUTHOR RESPONSE:** We have thoroughly reviewed the PLOS ONE style templates and confirm that the manuscript now fully complies with all formatting guidelines, including file naming conventions. All sections (title page, main text, references, and figures) have been adjusted to meet the journal's requirements.

2. In your Methods section, please provide additional information regarding the permits you obtained for the work. Please ensure you have included the full name of the authority that approved the field site access and, if no permits were required, a brief statement explaining why.

**AUTHOR RESPONSE:** In the Methods section (specifically in the "Samples collection" subsection), we have added the following clarification:
"This study was conducted with approval from the Chengdu Center for Disease Control and Prevention. No endangered or protected species or human subjects were involved in this study." (**Line 131-133**)

3. When completing the data availability statement of the submission form, you indicated that you will make your data available on acceptance. We strongly recommend all authors decide on a data sharing plan before acceptance, as the process can be lengthy and hold up publication timelines. Please note that, though access restrictions are acceptable now, your entire data will need to be made freely accessible if your manuscript is accepted for publication. This policy applies to all data except where public deposition would breach compliance with the protocol approved by your research ethics board. If you are unable to adhere to our open data policy, please kindly revise your statement to explain your reasoning and we will seek the editor's input on an exemption. Please be assured that, once you have provided your new statement, the assessment of your exemption will not hold up the peer review process.

**AUTHOR RESPONSE:** We confirm our commitment to PLOS ONE's open data policy. Our complete dataset will be deposited in the Supporting information (S2 Table. The raw data needed to replicate the findings of this study) of this article upon acceptance.

4. Please ensure that you refer to Figure 5 in your text as, if accepted, production will need this reference to link the reader to the figure.

**AUTHOR RESPONSE:** We confirm that Figure 5 is now correctly cited in the text, first appearing in the Results section (specifically in the “Application of wastewater surveillance for early warning of COVID-19 outbreak in Chengdu 2023 FISU world university games village” subsection). (**Line 322**)

**Author’s** **Responses to Review Comments**

**Comments from Reviewer #1:**

1. Add the specific objectives of the study.

**AUTHOR RESPONSE:** Many thanks for the Reviewer’s valuable suggestion.We have carefully revised the Abstract and Introduction sections to explicitly state the specific objectives of the study. It is as follows:

Corresponding update in Abstract (Objective section): "This study was conducted to enhance conventional epidemiological surveillance by implementing city-wide wastewater monitoring of SARS-CoV-2 RNA. The research aimed to develop a quantitative model for estimating infection rates and to compare these predictions with clinical case data. Furthermore, this wastewater surveillance was utilized as an early warning system for potential COVID-19 outbreaks during a large international event, the Chengdu 2023 FISU Games)." (**Line 21-26**)

Revised text in Introduction section: "The specific objectives of this study were threefold: (1) To establish a city-wide wastewater surveillance system in Chengdu as a complementary tool to traditional epidemiological monitoring, enabling real-time tracking of SARS-CoV-2 RNA dynamics across nine wastewater treatment plants serving 15.2 million residents. (2) To develop a quantitative model correlating wastewater viral concentrations with clinical case data, aiming to estimate the true scale of COVID-19 infections, including unreported cases, and to evaluate discrepancies between wastewater-based predictions and official nucleic acid testing records. (3) To implement targeted wastewater surveillance in the Chengdu 2023 FISU World University Games village as an early warning system for potential outbreaks, ensuring the timely detection of outbreaks during the international event." (**Line 115-127**)

2. This study employed wastewater based epidemiology (WBE), utilizing samples collected twice a week from nine wastewater treatment plants that serve 66.1% of Chengdu's residents, totaling 15.2 million people. A complete paragraph on the choice of stations with a clarification of the sampling plan is necessary. Why not choose a number of stations covering 80% of the population of Chengdu? Explain the details of the criteria for choosing these stations, as the size of the population must be part of a relevant multi-criteria set.

**AUTHOR RESPONSE:** We sincerely appreciate your constructive feedback on our study design. Below, we provide a detailed clarification regarding the selection criteria for wastewater treatment plants and the rationale for population coverage.

(1) The nine WWTPs were systematically selected based on a hierarchical evaluation of the following criteria:

First, regarding population coverage and density prioritization, the six urban districts covered by these WWTPs (Qingyang, Jinjiang, Wuhou, Jinniu, Chenghua, and the High-Tech Zone) constitute socio-economic core of Chengdu, serving 15.2 million residents (66.1% of the city’s total population). These districts exhibit a population density of 15,854 persons/km², 6.2 times higher than that of suburban areas. High-density urban populations are epidemiologically critical for wastewater-based surveillance, as they enhance community transmission signals and reduce spatial dilution effects.

Second, regarding geographic representativeness, the selected districts include a variety of community typologies, such as residential areas, commercial hubs, academic clusters, and transit centers, reflecting diverse population mobility patterns. This diversity enhances the generalizability of viral trend detection across urban demographics.

Third, concerning infrastructure compatibility, all nine WWTPs are equipped with standardized inlet structures, automated sampling systems, and centralized locations within a 1-hour transport radius from our laboratory. This arrangement ensures strict adherence to sample preservation protocols (4°C storage) and delivery within 2 hours post-collection. In contrast, suburban WWTPs (serving the remaining 17 districts) are geographically dispersed, with average transport times exceeding 2 hours, posing risks of analyte degradation, such as RNA instability.

Fourth, regarding data continuity and quality assurance, these WWTPs have participated in municipal environmental monitoring programs, and can provide datasets on daily flow rates and NH4-N concentrations. This data facilitates baseline normalization and enhances temporal trend analysis.

(2) While expanding coverage to 80% would necessitate the addition of several suburban WWTPs, this approach was considered suboptimal for the following reasons:

First, suburban sewer networks exhibit lower collection efficiency due to septic tank leakage and incomplete pipe connectivity, potentially biasing viral load measurements. Second, 70% of suburban WWTPs exceed the 2-hour cold chain transport threshold, increasing RNA degradation risks.

As suggested, we will add a dedicated subsection “Wastewater treatment plants (WWTPs) selection” to elaborate points mentioned above.

“The selection criteria for the WWTPs were based on the following factors: 1) serving high-density urban populations; 2) encompassing diverse community typologies, including residential areas, commercial centers, academic institutions, and transit hubs; 3) featuring standardized inlet structures, automated sampling systems, and centralized locations within a one-hour transport radius from our laboratory; and 4) having participated in municipal environmental monitoring programs and being able to provide datasets on daily flow rates and NH4-N concentrations. Consequently, nine WWTPs serving the entire population of Chengdu's main urban area were selected for sewage sample collection.” (**Line 133-141**)

3. Can you explain why Spearman was chosen instead of Pearson?

**AUTHOR RESPONSE:** Thank you for raising this important methodological question. We chose Spearman’s rank correlation over Pearson’s correlation for several reasons that align with the nature of our data. Both variables, weekly viral load in wastewater and registered COVID-19 cases, exhibited non-normal distributions, as confirmed by Shapiro-Wilk normality tests (p < 0.05 for both variables). Pearson’s correlation assumes linearity and normality, which were not met in our dataset. In contrast, Spearman’s method is non-parametric and does not require these assumptions, making it robust against skewed distributions and outliers.

4. From the third week to the twenty-fourth week of 2023, the weekly median concentration of SARS-CoV-2 RNA fluctuated, starting at 16.94 copies/ml in the third week, decreasing to 1.62 copies/ml by the fifteenth week, then gradually rising to a peak of 41.27 copies/ml in the twentieth week, before ultimately declining to 8.74 copies/ml by the twenty-fourth week. In fact, various parameters (flow rate from stations, information on hydraulic management of networks, chemical parameters, etc.) are most often integrated in order to correct these fluctuations as effectively as possible. Why didn't you take into account the data on fluctuation factors? What have you done statistically to smooth out the observed fluctuations?

**AUTHOR RESPONSE:** We appreciate the reviewer’s insightful questions regarding the observed fluctuations in SARS-CoV-2 RNA concentrations and the integration of fluctuation factors. Below, we address these points in detail:

First, in this study, we incorporated two key parameters to account for variability in wastewater measurements.

(1) Daily flow rates. The weighted average viral load for the city was calculated using the formula

Weighted average viral load _city_ = $\frac{\sum_{i=1}^{9} C\text{n}\times Q\text{n}}{\sum_{i=1}^{9} Q\text{n}}$, Where Cn represents the concentration of SARS-CoV-2 RNA in wastewater at WWTPn (copies/mL), and Qn represents the daily 24-hour volume of wastewater at WWTPn (mL/day).

(2) NH4-N concentrations. Population size in each WWTP catchment area was estimated using NH4-N data (4.2 g/person/day), which indirectly accounts for hydraulic contributions from the served population.

However, we acknowledge the additional parameters mentioned by the reviewer, such as the hydraulic management of networks, pH, temperature, and finer temporal resolution of flow rates, which were not explicitly incorporated into the model. This decision was primarily driven by limitations in data accessibility, such as the unavailability of detailed hydraulic data from municipal systems, and our focus on establishing a baseline correlation between wastewater viral loads and clinical cases in the post-epidemic era. Future studies will prioritize the integration of these factors to enhance the model's robustness.

Second, to address variability in the raw viral concentration data, we implemented the following approaches.

(1) Weekly median aggregation and weighted average viral load of city. Raw daily viral concentrations were aggregated into weekly medians and weighted average viral load of city to reduce noise from day-to-day sampling variability.
(2) Spearman’s correlation analysis. This non-parametric method was chosen to assess trends between wastewater viral loads and clinical cases, as it is less sensitive to outliers and non-normal distributions compared to Pearson’s correlation.
(3) Monte Carlo simulation. Uncertainty in back-calculating infection rates was addressed through 10,000 iterations, incorporating variability in viral shedding rates and population estimates (NH4-N-derived). The bootstrapped confidence intervals (P5–P95) reflect the stochastic nature of these parameters.

While these steps have mitigated some fluctuations, we acknowledge that additional smoothing techniques, such as moving averages and autoregressive models, or advanced normalization using hydraulic and chemical parameters could further enhance trend resolution. We will explore these methods in future iterations of the model.

**Comments from Reviewer #2:**

1. It is unclear why samples were taken from specific manholes in addition to WWTPs?

**AUTHOR RESPONSE:** We appreciate the reviewer’s insightful question. While the nine wastewater treatment plants (WWTPs) provided city-wide coverage (66.1% of Chengdu’s population), the two manholes were strategically selected to monitor exclusively the Chengdu 2023 FISU World University Games village. This village housed a closed, high-density international population during the event. Sampling from manholes allowed us to detect localized outbreaks within this specific community without dilution from broader urban wastewater flows. Rapidly identify potential SARS-CoV-2 transmission among athletes and staff, enabling immediate containment measures if needed. Additionally, the absence of significant viral concentration changes before and after check-in (Results section) confirmed no outbreaks occurred, aligning with clinical data from the symptom monitoring system. In summary, the inclusion of manhole sampling was essential to fulfill our dual aims of city-wide epidemiological modeling and targeted early-warning surveillance during a critical public health event.

2. In Abstract, 66% of population is served by nine WWTPs but in Line 106: entire population is served.

**AUTHOR RESPONSE:** We sincerely appreciate your careful attention to this detail. The apparent discrepancy arises from differences in the spatial scope of the referenced populations, which we clarify below:

In the Abstract and main text, the statement "nine wastewater treatment plants (WWTPs) serve 66.1% of Chengdu’s residents (15.2 million people)" refers to the proportion of Chengdu’s total municipal population covered by the selected WWTPs. In Line 106 (Methods section), the phrase "serving the entire population of Chengdu’s main urban area" specifically pertains to the demographic completeness within Chengdu’s core urban zone. The nine WWTPs were selected to represent 100% of the main urban area’s population, which constitutes 66.1% of Chengdu’s total municipal population (including suburban and rural regions).

To avoid ambiguity, we revised the sentence in the Methods to explicitly state: “Consequently, nine WWTPs serving the entire population of Chengdu’s main urban area (15.2 million residents, which represents 66.1% of the city’s total population) were selected for sewage sample collection.” (**Line 140-142**)

3. It is unclear where samples are collected? the influent or effluent of WWTPs.

**AUTHOR RESPONSE:** We thank the reviewer for highlighting this important methodological clarification. All wastewater samples in this study were collected exclusively from the influent (incoming raw sewage) of the nine WWTPs. To eliminate any ambiguity, we have revised the Methods section to explicitly state:

“A composite sewage sample of 2.4L was collected from the influent channels of each WWTP at one-hour intervals over a 24-hour period, from 10:00 AM to 10:00 AM the following day, during each sampling occasion.” (**Line 151-154**)

4. Since 2.4-L sample is collected during 24 hours, its biological content is doubtful. Its viral content would be deteriorated from the early auto-sampling. Each sample mainly represents the viral content of late sampling (e.g. 8-10 AM) than its previous day..

**AUTHOR RESPONSE:** We sincerely appreciate the reviewer’s insightful comments regarding the potential temporal degradation of viral RNA in 24-hour composite wastewater samples. We acknowledge the potential for RNA degradation during prolonged sampling. To mitigate this, our auto-samplers (equipped with peristaltic pumps) were programmed to collect 100 mL aliquots hourly into sterile containers pre-chilled at 4℃. Each aliquot was immediately stored in the sampler’s refrigerated compartment (4℃) to minimize RNA degradation. After the 24-hour collection period, the composite sample was transported to the laboratory within 1 hour under cold-chain conditions (4℃) and processed immediately. Studies have demonstrated that SARS-CoV-2 RNA in wastewater remains stable for up to 24 - 48 hours at 4℃ (Sala-Comorera L, Reynolds LJ, Martin NA, O'Sullivan JJ, Meijer WG, Fletcher NF. Decay of infectious SARS-CoV-2 and surrogates in aquatic environments. Water Res. 2021 Aug 1;201:117090. doi: 10.1016/j.watres.2021.117090.). Thus, the 24-hour period (10:00 AM to 10:00 AM) aligns with typical human activity cycles (e.g., morning excretion peaks), ensuring a representative average of daily viral shedding. Our future studies will incorporate RNase inhibitors during collection to enhance RNA stability.

5. Usually, there is a hydraulic retention time between the residential areas and WWTPs in wastewater collection systems. Authors should clarify with proper data how much time it takes on average for the sewage to travel from these areas to 9 WWTPs. It affects both the viral content and early warning systems.

**AUTHOR RESPONSE:**  We appreciate the reviewer’s insightful comments regarding the hydraulic retention time (HRT) in wastewater collection systems and its impact on viral content stability. We provide clarifications based on available data and engineering design standards.

The 9 selected WWTPs primarily serve high-density urban districts in Chengdu, including Wuhou, Qingyang, Jinniu, Chenghua, Jinjiang, and High-Tech Zone. Urban WWTPs in China generally cover a service radius of 5 km-15 km, depending on topography and pipeline layout. The average HRT from residential areas to WWTPs depends on pipe flow velocity and network complexity. Municipal sewage systems in China typically operate at 1.0 m/s under gravity flow. For a 5 km pipeline, HRT = distance/velocity=5000 m/1m/s=1.39 hours. For a 15 km pipeline, HRT = distance/velocity=15000 m/1m/s=4.17 hours. For average 8 km pipeline, HRT = distance/velocity=8000 m/1m/s=2.22 hours. In the Methods section (Wastewater treatment plants (WWTPs) selection), we added the sentence “Urban WWTPs in Chengdu generally serve a radius of 5 to 15 kilometers, with an average of 8 kilometers. The average hydraulic retention time (HRT) from residential areas to WWTPs is influenced by pipe flow velocity and the complexity of the network. Municipal sewage systems in China typically operate at a flow velocity of 1.0 m/s under gravity flow conditions. For an average 8-kilometer pipeline, the HRT can be calculated as follows: HRT = distance/velocity = 8000 m / 1 m/s = 8,000 seconds, or approximately 2.22 hours.” (**Line 142-149**)

6. Line 194: It is confusing. authors are pointing to 9 WWTPs, but there is a 10th WWTP. Again in Line 194: The flow of 9th WWTP equals the 10th WWTP: 996.7. This confusion continues in Line 201: 10th-1 and 10th-2 WWTPs?

**AUTHOR RESPONSE:** We appreciate the reviewer’s careful reading and apologize for the confusion caused by the nomenclature of WWTPs. Our study analyzed 9 WWTPs in total. These are systematically numbered as the "3rd to 10th WWTPs" based on their administrative codes within Chengdu’s urban area. The apparent inconsistency arises because the original 10th WWTP was subdivided into two independent facilities (referred to as 10th-1 and 10th-2) prior to our study. Thus, despite the numbering extending to "10th," the total remains 9 distinct plants.

The statement “the flow of 9th WWTP equals the 10th WWTP: 996.7” was an error in phrasing. The correct interpretation is: the 9th WWTP has the highest flow rate (996.7 thousand m³/d) among all 9 plants, and the subdivided 10th-1 and 10th-2 WWTPs have lower individual flows (48.1 and 249.2 thousand m³/d, respectively). We have revised the text to consistently use 10th-1 and 10th-2 (instead of "10th WWTP") to avoid ambiguity. This subdivision reflects operational separation in the field, as the two facilities serve distinct catchment zones despite sharing the same administrative code. We have revised the manuscript as follows:

“Wastewater samples were collected from nine WWTPs (numbered numbered 3 through 10 according to Chengdu’s administrative codes). The 9th WWTP exhibited the highest average daily flow (996.7 thousand m³/d), while the subdivided 10th-1 and 10th-2 WWTPs had flows of 48.1 and 249.2 thousand m³/d, respectively.” (**Line 241-244**)

7. Line 202 and Table 1: Why the sampling dates are different to the study period? wasn't it presumed that sampling is continued to June 15? Table 1 is confusing in numbering the WWTPs and the time of samples.

**AUTHOR RESPONSE:** We sincerely thank the reviewer for highlighting this ambiguity. We provide the following clarifications:

First, the overall study period spanned from January 18 to June 15, 2023, during which continuous wastewater sampling was conducted twice weekly (Mondays and Thursdays) at all nine WWTPs. The dates listed in Table 1 (e.g., January 18 and February 20) indicate the initial sampling date for each WWTP, not the entire sampling duration. For example: The 4th, 10th-1, and 10th-2 WWTPs initiated sampling on January 18, while other plants began on February 20 due to logistical coordination with local authorities. All WWTPs continued sampling until June 15, with frequencies and total sample numbers (34 or 43 per plant) reflecting this full period (see "Samples Number" in Table 1).

We have revised the manuscript as follows: "Sampling began on January 18, 2023, for the 4th, 10th-1, and 10th-2 WWTPs, and on February 20 for the remaining facilities. Twice-weekly collections continued consistently until June 15, 2023." (**Line 151-153**)

Second, to resolve confusion, we have renamed the column "Sampling Date" to "Initial Sampling Date" to emphasize continuity. We also added a footnote to Table 1: "Initial sampling dates indicate the first collection day for each WWTP; sampling continued twice weekly until June 15, 2023. The total sample numbers reflect cumulative collections over the entire study period." (**Table 1**)

8.How much is it possible that the daily sewage of some individuals are considered in two or more WWTPs? I mean if someone is infected and lives in Place A, but works in Place B, its wastewater would enter two WWTPs. How much is the rate of these individuals?

**AUTHOR RESPONSE:** We sincerely appreciate the reviewer’s insightful question regarding the potential overlap of individuals contributing sewage to multiple WWTPs. Below, we address this concern by integrating Chengdu’s urban characteristics.

The nine WWTPs selected in this study serve distinct geographic zones within Chengdu’s main urban area, with an average service radius of 8 km (range: 5–15 km). These zones are planned based on municipal functional divisions (e.g., residential clusters, commercial centers, academic campuses), minimizing overlaps in their catchment areas. While cross-WWTP mobility (e.g., individuals residing in Zone A and working in Zone B) is possible, such cases are likely limited due to two reasons. First, residential and commercial zones in Chengdu are spatially clustered, with most daily commutes occurring within the same administrative district (average commute distance: 9.7 km). However, there was no report on the rate of individuals living and working in different zones. Second, infected individuals typically shed viral RNA at peak levels during the symptomatic phase (1 to 5 days post-onset), when they are more likely to stay at home, thereby reducing cross-WWTP contamination.

9. Line 213-217: how much is the accuracy of experiments? It should be clarified, particularity when you are pointing to small variations (83%-100%).

**AUTHOR RESPONSE:** We sincerely appreciate the reviewer’s insightful question regarding the accuracy of our experiments. Below, we provide a detailed clarification of the experimental accuracy.

Our RT-qPCR protocol was rigorously validated to ensure reliability. The SARS-CoV-2 nucleic acid detection kit (Liferiver Bio-Tech) targets two conserved regions (ORF1ab and N genes) with established specificity (no cross-reactivity with other coronaviruses or human microbiota) and a detection limit of 10 copies/mL, as validated in previous studies (Li Y, Du C, Lv Z, Wang F, Zhou L, Peng Y, et al.. Rapid and extensive SARS-CoV-2 Omicron variant infection wave revealed by wastewater surveillance in Shenzhen following the lifting of a strict COVID-19 strategy. Sci Total Environ 2024;949:175235). More over, all samples and controls were tested in triplicate, with intra-assay coefficients of variation (CV) for Ct values ≤ 5%. Additionally, a plate-specific standard curve (R² > 0.99, efficiency: 90%–110%) was generated using serially diluted reference materials (GBW(E)091133) to ensure quantitative accuracy. In conclusion, our methodology demonstrates high accuracy and reproducibility, with variations in positivity rates reflecting true epidemiological shifts and near-limit-of-detection stochasticity.

We have revised the manuscript to explicitly address these points in the Methods:

“Diethylpyrocarbonate (DEPC) treated water as negative template controls (NTCs), and DNA plasmids as positive control were included for every batch, and each sample and control were detected in triplicate, with intra-assay coefficients of variation for Ct values ≤ 5%. A sewage sample would be classified as“positive” only if the Ct values of either the ORF1ab or N genes were below 43, and its virus concentration was calculated according to the plate-specific standard curve (R² > 0.99, efficiency: 90%–110%) established by ten-fold serial dilution of the reference materials (GBW(E)091133, Bdsbiotech, Guangzhou, PRC) from 10^6^ to 10^1^ copies/ml.” **(Line 191-197)**

10.I assumed that cases point to new daily and weekly cases. But why in Figure 4, the number of cases are steady for some weeks?

**AUTHOR RESPONSE:** We sincerely appreciate the reviewer's insightful observations. The relatively stable case numbers during specific weeks, as illustrated in Figure 4, can be attributed to the unique epidemiological context of the FISU World University Games Village and our comprehensive prevention measures. First, during the Games period (July 20-August 11), we implemented routine symptom monitoring and rapid response protocols. These measures effectively suppressed secondary transmissions, which accounts for the stable case numbers (23 total cases) despite intermittent viral detection in wastewater. Second, wastewater surveillance detected low-level viral signals (≤0.56 copies/mL) 11 days prior to the initiation of clinical case reporting, demonstrating its capability as an early warning system. However, the stabilized case numbers reflect the success of subsequent containment measures in preventing exponential growth, even as wastewater monitoring continued to detect baseline viral shedding. Third, the apparent stabilization in case counts results from the aggregated reporting of sporadic, isolated cases that did not escalate into outbreaks. Higher temporal resolution data (e.g., daily case curves) revealed minor fluctuations that corresponded to wastewater trends.

11.Figure lack proper legends.

**AUTHOR RESPONSE:** We sincerely appreciate the reviewer's insightful observations. We have added the appropriate legends to the figures.

12.Authors must clarify their specific innovation of their research, particularly in comparison with similar studies in the same area (like reference No.16).

**AUTHOR RESPONSE:** We sincerely thank the reviewer for raising this critical point. Our study advances WBE for SARS-CoV-2 surveillance in two key innovative aspects, differentiating it from prior work such as Reference 16 and similar studies. Below, we clarify these innovations:

First, we developed and validated a quantitative model for estimating infection rates. While Reference 16 and other WBE studies focus on qualitative or semi-quantitative correlations between wastewater viral loads and clinical case trends, our work introduces a robust quantitative model that estimates infection rates and predicts actual infected populations. This addresses a critical gap in translating wastewater data into actionable public health metrics. We incorporated hydrological, demographic, and viral shedding parameters to calculate daily infection rates, which range from 0.012% to 3.27%, and cumulative infections, totaling 2.26 million individuals—33 times higher than registered cases. This model accounts for uncertainties (e.g., P5-P95 intervals) and provides a scalable framework for resource allocation, unlike Reference 16, which primarily correlates trends without quantifying hidden transmission. The strong correlation (Spearman’s r = 0.93, P < 0.001) between modeled estimates and clinical data further validates its reliability.

Second, we implemented wastewater surveillance as a real-time early warning system for large-scale international events. To our knowledge, no prior WBE study, including Reference 16, has demonstrated the operational deployment of wastewater surveillance for preemptive outbreak prevention during a densely populated international event such as the FISU World University Games. Our dual surveillance system, which combines wastewater analysis and symptom monitoring at the Games Village, confirmed the absence of outbreaks despite the presence of transient viral signals (≤0.56 copies/mL). This finding underscores the utility of WBE in safeguarding high-risk gatherings. In contrast, Reference 16 focuses on city-scale monitoring rather than event-specific, real-time risk mitigation.

We revised the article to explicitly contrast our methodological and operational advances with those in Reference 16 and similar studies, emphasizing: (1) the quantitative model's ability to estimate infection rates and hidden transmission, and (2) the novel application of WBE for preemptive outbreak prevention at large international events. (**Line 420-422**)

13.Line 291: r = 0.98 or r2?

**AUTHOR RESPONSE:** It was spearman’s r (spearman’s r =0.93). We have revised this in the article **(Line 357-358)**.

14.Regarding Line 304-306, it is necessary for authors to report the status of WWTPs.

**AUTHOR RESPONSE:** We have reported the status of WWTPs, added the catchment zone, temperature (℃), total suspended solids (mg/L), and chemical oxygen demand (mg/L) in Table 1 (The characteristics of nine WWTPs).

15.I wonder how much personal curation would affect the results? I mean people have gradually learned how to cure themselves without going to hospitals for COVID-19. It may significantly change the rates and correlation.

**AUTHOR RESPONSE:** We thank the reviewer for highlighting this important issue regarding the potential impact of self-management behaviors (e.g., reduced hospital visits and increased reliance on at-home care) on the correlation between wastewater surveillance and reported clinical cases. We recognize that changes in healthcare-seeking patterns may affect official case reporting rates. However, our analysis indicates that wastewater surveillance continues to serve as a reliable indicator of community-level infection trends, even in the context of evolving clinical reporting practices. Below, we address this concern by presenting our findings:

First, despite the potential underreporting of clinical cases due to self-management (e.g., reduced nucleic acid testing), wastewater SARS-CoV-2 RNA concentrations (Fig. 2-3) exhibited a strong temporal correlation with registered cases (P < 0.001). This alignment persisted throughout both the rising and falling phases of the epidemic, including the post-"May Day" surge and the subsequent decline. The sustained correlation suggests that wastewater data accurately reflects true infection dynamics, independent of healthcare-seeking behavior.

Second, our model estimated that 2.26 million individuals (95% CI: 0.85–3.93 million) were infected during the study period, far exceeding the 68,190 registered cases, representing a 33-fold difference. This discrepancy underscores significant underreporting, likely driven by asymptomatic or mild infections and self-management. Importantly, wastewater trends captured this "hidden" transmission, demonstrating their utility for tracking true prevalence even when clinical reporting is incomplete.

While our study did not directly measure self-management behaviors (e.g., surveys on healthcare avoidance), the high sensitivity of wastewater surveillance and its correlation with clinical trends suggest that these behaviors did not disconnect wastewater data from actual infection rates. Future research could incorporate anonymized mobility data or health-seeking surveys to further validate this relationship.

In conclusion, although self-management may lead to a decrease in clinical reporting rates, wastewater surveillance offers a passive, population-level indicator of SARS-CoV-2 transmission that remains resilient to changes in healthcare utilization. Our findings underscore its significance for public health decision-making, especially in contexts characterized by underreporting.

16.Conclusion needs some major revision to illustrate the whole findings of research.

**AUTHOR RESPONSE:** Thank you for your valuable comment. We have revised the conclusion, which is as follows:

“This study establishes WBE as a crucial tool for tracking SARS-CoV-2 during Chengdu's post-pandemic phase. Monitoring nine wastewater treatment plants that serve 15.2 million residents revealed a second epidemic wave five months after the lifting of restrictions, which strongly correlated with clinical trends. Model estimates indicated approximately 2.26 million infections, 33 times higher than the reported cases, peaking at an infection rate of 3.27%. WBE provided an early warning at the FISU Games Village, aligning with symptom monitoring efforts. Despite the limitations of sampling and back-calculation models, including fecal and urine viral shedding rates, RNA degradation, and in-sewer dilution, WBE has proven to be effective in identifying hidden transmission and guiding public health responses” . **(Line 444-454)**

17.The whole results require statistical attachments about the average, standard errors, and the significance of difference between outbreaks.

**AUTHOR RESPONSE:** We appreciate the reviewer’s suggestion to enhance the statistical rigor of our findings. In the revised manuscript, we have included the average, standard error, and significance levels. **(Line 311-325)**.

**Comments from Reviewer #3:**

1. (Line 1): The title is informative but could be more precise. Consider specifying the study's key objective, such as "Wastewater Surveillance as a Predictive Tool for COVID-19: A Case Study in Chengdu."

**AUTHOR RESPONSE:** We sincerely appreciate the reviewer's insightful suggestion to enhance the clarity and precision of our manuscript's title. After careful consideration, we agree that specifying the study's key objective and geographic focus would better reflect the manuscript's contributions. We have revised the title to:

"Wastewater Surveillance as a Predictive Tool for COVID-19: A Case Study in Chengdu". (**Line 4-5**)

2. (Line 19-21): The abstract mentions a "quantitative wastewater surveillance model," but does not briefly describe how it works. Adding a sentence explaining the core method used (e.g., back-calculation) would improve clarity.

**AUTHOR RESPONSE:** We thank the reviewer for this valuable suggestion to enhance methodological transparency. As requested, we have added the following sentence to the abstract to clarify the core back-calculation approach: "A model employs back-calculation of SARS-CoV-2 infections by integrating wastewater viral load measurements with human fecal and urinary shedding rates, as well as population size estimates derived from NH4-N concentrations, utilizing Monte Carlo simulations to quantify uncertainty." (**Line 31-34**)

3. (Line 37-38): The phrase "which is roughly 33 times the number of registered cases" is important but should highlight the implications, which is does this suggest significant underreporting?

**AUTHOR RESPONSE:** We thank the reviewer for highlighting the necessity of explicitly interpreting the 33-fold discrepancy between wastewater-predicted infections and officially registered cases. As suggested, we have revised the Discussion section to emphasize its implications.

“The estimated peak infection burden from wastewater was approximately 33 times higher than the officially reported cases during the same period. This substantial disparity strongly indicates significant underreporting following China's transition to Category B management, which excluded both untested symptomatic individuals and asymptomatic carriers from surveillance systems. This underdetection aligns with global observations that wastewater surveillance captures 11 times more COVID-19 cases than clinical reporting [34], underscoring the critical role of wastewater epidemiology in complementing conventional surveillance.” (**Line 383-391**)

4. (Line 62-66): "It does not imply that COVID-19 is no longer a global health threat." This sentence is clear, but it would be stronger if linked to the need for alternative surveillance systems, reinforcing the relevance of the study.

**AUTHOR RESPONSE:** Thank you for your constructive feedback. We have revised the sentence to explicitly emphasize the necessity of alternative surveillance systems like WBE to address ongoing COVID-19 risks. The revised text in the Introduction now reads: “Additionally, conventional sentinel surveillance, which relied on clinical testing and hospital reporting, faced limitations in capturing true infection dynamics, particularly under reduced testing frequency and asymptomatic transmission. It is crucial to utilize wastewater-based epidemiology (WBE) as a vital complementary tool to maintain and enhance epidemic surveillance, effectively monitoring virus mutations and the progression of the epidemic. ” (**Line 80-85**)

5. (Line 75-83): The section discussing China’s monitoring system could be improved by briefly explaining how WBE overcomes the drawbacks of traditional methods.

**AUTHOR RESPONSE:** Thank you for your valuable suggestion. We have revised the section to explicitly highlight how WBE addresses the drawbacks of traditional methods. The updated text in the Introduction now reads: “WBE can overcome the limitations of traditional methods, such as the need for resource-intensive testing campaigns, the underreporting of asymptomatic or mild cases, and the risks of transmission associated with mass gatherings. It achieves this by providing real-time, population-level data without requiring individual participation. By analyzing SARS-CoV-2 RNA concentrations in sewage, WBE facilitates continuous monitoring of infection trends across entire communities, including unreported cases. This approach offers a more comprehensive and unbiased assessment of epidemic progression [13–15] ”. (**Line 104-111**)

6. (Line 85-95): The rationale for WBE is clear, but a brief mention of the key studies supporting its use would strengthen the argument.

**AUTHOR RESPONSE:** Thank you for your valuable suggestion. We have added the key study in the Introduction. (**Lines 111-113**)

7. (Line 117-118): Consider explaining why NH4-N concentration was chosen to estimate population size.

**AUTHOR RESPONSE:** NH4-N, the hydrolysis product of urea in human urine, is the most indicative parameter among sewage characteristics for reflecting human metabolism. Its concentration fluctuates in response to changes in population size. (Reference 1: Been F, Rossi L, Ort C, Rudaz S, Delémont O, Esseiva P. Population normalization with ammonium in wastewater-based epidemiology: application to illicit drug monitoring. Environ Sci Technol. 2014 Jul 15;48(14):8162-9. doi: 10.1021/es5008388. Reference 2: Degao W. Wastewater-based epidemiology. Beijing: Science Press; 2018. )

8. (Line 145-146): The statement about controls should explicitly state whether negative controls were included.

**AUTHOR RESPONSE:** We sincerely appreciate the reviewer’s insightful feedback regarding the statement of negative controls. We have added the following statement in the Methods section: “Diethylpyrocarbonate (DEPC) treated water as negative template controls (NTCs), and DNA plasmids as positive control were included for every batch, and each sample and control were detected in triplicate, with intra-assay coefficients of variation for Ct values ≤ 5%” . (**Line 191-194**)

9. (Line 148-149): The cut-off Ct value of 43 seems high. Justify why this threshold was used.

**AUTHOR RESPONSE:** Thank you for raising this important point. The Ct cutoff of 43 for defining a sewage sample as "positive" was carefully selected based on the following rationale. First, the SARS-CoV-2 nucleic acid detection kit (Liferiver Bio-Tech) used in this study recommends a Ct threshold of ≤ 43 for positive identification, as specified in its protocol. This threshold aligns with the kit’s validated limit of detection for low viral RNA concentrations in clinical and environmental matrices. Second, all negative controls (DEPC treated water) consistently yielded Ct values >43 (undetected) across all batches, confirming the absence of cross-contamination and supporting the specificity of this threshold. Third, wastewater samples inherently contain PCR inhibitors and fragmented RNA, which can reduce amplification efficiency. Extending the cycle threshold to 43 enhances sensitivity for detecting trace SARS-CoV-2 signals in sewage.

10. (Line 163-164): The assumed stool shedding rate is taken from previous studies but should be validated against recent WBE literature.

**AUTHOR RESPONSE:** We appreciate the reviewer's insightful comment regarding the validation of SARS-CoV-2 RNA shedding rates in stool. In recent WBE literature (Li Y, Du C, Lv Z, Wang F, Zhou L, Peng Y, et al.. Rapid and extensive SARS-CoV-2 Omicron variant infection wave revealed by wastewater surveillance in Shenzhen following the lifting of a strict COVID-19 strategy. Sci Total Environ 2024;949:175235), it reported the Omicron variant-specific faecal viral loads of participants in Shenzhen during the acute period ranged from 4.2 to 10.6 log10 copies/g (mean = 7.8 log10 copies/g). In our study, the stool in wastewater not only from patients in the acute phase, but also from those in the convalescent phase and asymptomatic phase. Thus, the amount of SARS-Cov-2 RNA shedding in the stool of an infected person was assumed 6.23 log10 copies/g (1.7*10^6 copies/g) which was within the range and very close to the average value of Omicron variant-specific faecal viral loads reported by Li et al.

11. (Line 174-178): The use of NH4-N to estimate population size is reasonable but should include a reference for its validity in previous studies.

**AUTHOR RESPONSE:** Thank you for raising this important point. We have added the reference (Been F, Rossi L, Ort C, Rudaz S, Delémont O, Esseiva P. Population normalization with ammonium in wastewater-based epidemiology: application to illicit drug monitoring. Environ Sci Technol. 2014 Jul 15;48(14):8162-9. doi: 10.1021/es5008388.) in the Methods. (**Line 222)**

12. (Line 224-225): The R² value (0.9633) indicates a strong correlation, but was the analysis adjusted for potential confounders such as changes in testing capacity?

**AUTHOR RESPONSE:** We appreciate the reviewer’s insightful questions regarding the analysis adjusted for potential confounders. We address these points in detail. First, Chengdu adhered to consistent national guidelines for nucleic acid testing throughout the study period (January–June 2023), with no abrupt changes in testing eligibility, criteria, or public reporting protocols. Second, the laboratory employed standardized RT-qPCR protocols. Strict quality controls (e.g., plate-specific calibration curves with R² > 0.99, triplicate measurements, and inter-assay CV < 5%) ensured consistent detection sensitivity across the study period, thereby eliminating technical variability as a confounding factor. Third, we took into account the fluctuations in daily flow rates, and the weighted average viral load for the city was calculated.

However, we acknowledge that other potential confounders, such as wastewater temperature, catchment size, chemical dosing, and the accuracy of clinically confirmed cases, may affect the correlation. We will investigate methods to adjust for these potential confounders in future analyses.

13. (Line 237-239): The statement that "approximately 2,258,245 individuals contracted COVID-19" is significant. Consider discussing uncertainty in this estimate (e.g., model limitations, underestimation due to viral degradation).

**AUTHOR RESPONSE:** We sincerely appreciate the reviewer’s valuable feedback regarding the uncertainty associated with our infection estimates. We agree that transparently addressing limitations is critical for interpreting the back-calculated case numbers. We have added the following limitations in the Discussion section: “Our back-calculation model did not account for the decay rate of the virus in sewers, which is influenced by wastewater temperature and the distance from the community to the WWTP. This oversight may have led to an underestimation of the viral load in wastewater and the actual number of infected individuals. Furthermore, the fecal and urine viral shedding loads in our model appear to vary between individuals and infection states, resulting in significant variability in the results. Further optimization of the modeling approach is necessary to enhance the accuracy of our findings.” (**Line 431-438**)

14. (Line 240-242): The infection rates estimated should be compared with independent estimates from seroprevalence or epidemiological models.

**AUTHOR RESPONSE:** Thank you for your valuable suggestion. We have revised the section to include a comparison with monitoring data from national sentinel hospitals and epidemiological models from Taiyuan. The updated text in the Discussion now reads: “The results are consistent with monitoring data from national sentinel hospitals, which indicated that the positive rate of COVID-19 among outpatients and emergency department cases was 2.5% (the proportion of influenza-like illness cases was 6.2%, and the positive rate of COVID-19 among influenza-like illness cases was 40.7%). However, these findings are inconsistent with the results from Taiyuan City, which utilized a dynamic model and suggested that the peak of infectious individuals during the second wave was only 0.88%.” (**Line 391-397**)

15. (Line 265-273): The comparison of wastewater data with symptom monitoring results is useful but lacks an explicit statement on whether the wastewater method was more effective.

**AUTHOR RESPONSE:** Thank you for highlighting this important point. Wastewater surveillance during a large international gathering proved effective and enhanced traditional monitoring methods. First, the viral loads in wastewater remained low, with no sustained spikes throughout the Games period, which was consistent with symptom monitoring and aligned with the absence of reported outbreaks. This consistency supports the use of wastewater data as a reliable proxy for population-level transmission intensity. Second, wastewater surveillance can detect infections regardless of testing compliance or symptom status (e.g., asymptomatic athletes). Third, wastewater surveillance is more cost-effective. We have included the explicit statement in the Discussion section: “Wastewater surveillance during a large international gathering was more effective and enhanced traditional monitoring methods”. (**Line 338-339**)

16. (Line 291-303): The discussion on correlation coefficients is strong but would benefit from a brief table summarizing the different correlation values observed in past studies.

**AUTHOR RESPONSE:** Thank you for your valuable suggestion. We have summarized the different correlation values observed in past studies in table s1.

Table s1. Summary of correlations in previous studies

| Location | Sampling site | Sampling mode | Sampling frequency | Population | Range of cases | Correlation R | P | Reference |
| --- | --- | --- | --- | --- | --- | --- | --- | --- |
| Canada | Hospitals | 24h composite | weekly | 2100 | 4-45 | 0.19-0.53 | 0.0009-0.2737 | 26 |
| Saudi Arabia | WWTPs | not mentioned | monthly | not mentioned | 57-160/100,000 people | 0.37-0.42 | Not significant | 27 |
| Canada | WWTPs | 24h composite | fortnightly | 1.1 millions | 10-60/100,000 people | 0.24-0.35 | ＜ 0.05 | 28 |
| Saudi Arabia | Hospitals | grab | 3-5 samples/week | 2884 | 73-236 | 0.21-0.24 | not mentioned | 29 |
| USA | Manholes in a university | grab | not mentioned | not mentioned | 269-526 | 0.48-0.51 | ＜ 0.001 | 30 |

17. (Line 311-319): The back-calculation model should be discussed in more detail. What are its key assumptions? How do those assumptions affect accuracy?

**AUTHOR RESPONSE:** We thank the reviewer for the opportunity to elaborate on the assumptions and limitations of our back-calculation model. The model relies on the following critical assumptions, which are grounded in published literature but inherently introduce uncertainty. First, the fixed fecal and urine shedding rates are derived from literature medians and do not account for individual variability (e.g., differences between asymptomatic and symptomatic shedding, as well as host immunity effects). This oversight could result in either overestimation or underestimation of the true shedding rates within the population. Second, the population served by the WWTP is calculated using NH4-N concentration. However, industrial and commercial NH4-N inputs (e.g., from fertilizers and food processing) or transient populations (e.g., tourists) could inflate population estimates, leading to an underestimation of infection rates. Third, the measured viral concentrations in wastewater directly reflect contributions from infected individuals, without explicit adjustments for RNA degradation or in-sewer dilution. RNA degradation may lead to an underestimation of true viral loads, particularly during warmer seasons or in areas with long hydraulic retention times. Additionally, dilution from stormwater inflow could further reduce measured concentrations, biasing infection estimates downward. We have included these details in the Discussion. (**Line 401-414**)

18. (Line 347-353): The conclusion is strong but should include a brief mention of key limitations, particularly regarding model assumptions

**AUTHOR RESPONSE:** Thank you for your valuable suggestion. We have revised the Conclusions: “Despite the limitations of sampling and back-calculation models, including fecal and urine viral shedding rates, RNA degradation, and in-sewer dilution, WBE has proven to be effective in identifying hidden transmission and guiding public health responses”. (**Line 451-454**)
