# Supplementary material for: Wastewater surveillance as a predictive tool for COVID-19: A case study in Chengdu
Source: PLoS One. 2025 May 28;20(5):e0324521. doi: 10.1371/journal.pone.0324521 (PMC12118905; doi:10.1371/journal.pone.0324521)
Supplement: Attachment — Submitted filename: Response_to_Reviewers_auresp_2.docx [file pone.0324521.s006.docx]

Dear Reviewers,

We greatly appreciate your comments and suggestions. In response to the reviewers' feedback, we have addressed each point raised by the academic editor and the reviewers. Additionally, we have made some changes to the manuscript, which are highlighted for your convenience.

**Author’s Responses to Academic Editor Comments**

Authors need to include the required discussion about the potential uncertainties embedded in their prediction as commented by the reviewer

**AUTHOR RESPONSE:** Thank you for your valuable feedback and the opportunity to revise our manuscript. We sincerely appreciate the time and effort invested by the reviewers in evaluating our work. As suggested, we have carefully revised the manuscript to address the comments raised by Reviewer #2. Specifically, in the Discussion section, we have added the following paragraph to discuss the potential uncertainties embedded in our prediction: “First, the fixed fecal and urine shedding rates are derived from literature medians and do not account for individual variability (e.g., differences between asymptomatic and symptomatic shedding, as well as host immunity effects). This oversight could result in either overestimation or underestimation of the true shedding rates within the population. Second, the population served by the WWTP is calculated using NH4-N concentration. However, industrial and commercial NH4-N inputs (e.g., from fertilizers and food processing) or transient populations (e.g., tourists) could inflate population estimates, leading to an underestimation of infection rates. Third, the measured viral concentrations in wastewater directly reflect contributions from infected individuals, without explicit adjustments for RNA degradation or in-sewer dilution. RNA degradation may lead to an underestimation of true viral loads, particularly during warmer seasons or in areas with long hydraulic retention times. Additionally, dilution from stormwater inflow could further reduce measured concentrations, biasing infection estimates downward.”. (**Line 404-417**)

Moreover, in the Discussion section, we have added the following paragraph to explicitly describe the sensitivity analyses performed to validate the robustness of our model: “In order to ensure the stability of the study results, we conducted sensitivity analyses. The faecal SARS-CoV-2 RNA shedding rate was chosen to be 82.4%; the daily faecal production per person was 128g, and the faecal NH4-N excretion varied ±10% from baseline values. The predicted infection rates calculated according to different parameters were similar (Table S3). Therefore, the results calculated by the model were reliable.” (**Line 423-428**)

**Author’s Responses to Journal Requirements**

Please review your reference list to ensure that it is complete and correct. If you have cited papers that have been retracted, please include the rationale for doing so in the manuscript text, or remove these references and replace them with relevant current references. Any changes to the reference list should be mentioned in the rebuttal letter that accompanies your revised manuscript. If you need to cite a retracted article, indicate the article’s retracted status in the References list and also include a citation and full reference for the retraction notice.

**AUTHOR RESPONSE:** We have carefully reviewed the reference list to ensure its completeness, accuracy, and correct.

**Author’s Responses to Additional Editor Comments**

Authors need to properly revise the manuscript considering the comments of second reviewer.

**AUTHOR RESPONSE:** Thank you for your valuable feedback and the opportunity to revise our manuscript. We sincerely appreciate the time and effort invested by the reviewers in evaluating our work. As suggested, we have carefully revised the manuscript to address the comments raised by Reviewer #2. Specifically, in the Discussion section, we have added the following paragraph to discuss the potential uncertainties embedded in our prediction: “First, the fixed fecal and urine shedding rates are derived from literature medians and do not account for individual variability (e.g., differences between asymptomatic and symptomatic shedding, as well as host immunity effects). This oversight could result in either overestimation or underestimation of the true shedding rates within the population. Second, the population served by the WWTP is calculated using NH4-N concentration. However, industrial and commercial NH4-N inputs (e.g., from fertilizers and food processing) or transient populations (e.g., tourists) could inflate population estimates, leading to an underestimation of infection rates. Third, the measured viral concentrations in wastewater directly reflect contributions from infected individuals, without explicit adjustments for RNA degradation or in-sewer dilution. RNA degradation may lead to an underestimation of true viral loads, particularly during warmer seasons or in areas with long hydraulic retention times. Additionally, dilution from stormwater inflow could further reduce measured concentrations, biasing infection estimates downward.”. (**Line 404-417**)

Moreover, in the Discussion section, we have added the following paragraph to explicitly describe the sensitivity analyses performed to validate the robustness of our model: “In order to ensure the stability of the study results, we conducted sensitivity analyses. The faecal SARS-CoV-2 RNA shedding rate was chosen to be 82.4%; the daily faecal production per person was 128g, and the faecal NH4-N excretion varied ±10% from baseline values. The predicted infection rates calculated according to different parameters were similar (Table S3). Therefore, the results calculated by the model were reliable.” (**Line 423-428**)

**Author’s** **Responses to Review Comments**

**Comments from Reviewer #1:**

I didn't see the answer to one of my concerns. In fact, various parameters (flow rate from stations, information on hydraulic management of networks, chemical parameters, etc.) are most often integrated in order to correct these fluctuations as effectively as possible. Why didn't you take into account the data on fluctuation factors? What have you done statistically to smooth out the observed fluctuations?

**AUTHOR RESPONSE:** We sincerely thank the reviewer for their constructive feedback and for reiterating the importance of addressing variability in wastewater viral load measurements. Below, we provide a point-by-point clarification and elaboration on our methodology to mitigate fluctuations and account for critical parameters.

1. Integration of fluctuation factors

We fully agree that parameters such as hydraulic management, chemical factors, and finer flow rate dynamics are vital for robust normalization. In our study, we prioritized two accessible and directly measurable parameters to correct fluctuations:

(1) Daily flow rate normalization. Viral loads were weighted by daily wastewater flow rates (Qn) to account for dilution/concentration effects. The formula:

Weighted average viral load city = $\frac{\sum_{i=1}^{9} C\text{n}\times Q\text{n}}{\sum_{i=1}^{9} Q\text{n}}$, adjusts for volumetric variations across sampling days and WWTPs, ensuring that high-flow days (which dilute viral RNA) do not artificially suppress population-level trends.

(2) NH_4_-N-Based Population Normalization. We estimated catchment populations using daily NH_4_-N loads (4.2 g/person/day). This approach indirectly accounts for hydraulic contributions from transient populations (e.g., commuters) and diurnal wastewater generation patterns, as NH_4_-N levels reflect real-time human fecal input.

However, we acknowledge the additional parameters mentioned by the reviewer, such as the hydraulic management of networks, pH, temperature, and finer temporal resolution of flow rates, which were not explicitly incorporated into the model. This decision was primarily driven by limitations in data accessibility, such as the unavailability of detailed hydraulic data from municipal systems, and our focus on establishing a baseline correlation between wastewater viral loads and clinical cases in the post-epidemic era. Future studies will prioritize the integration of these factors to enhance the model's robustness.

2. Statistical smoothing of fluctuations

To minimize noise and enhance trend resolution, we implemented:

(1) Weekly median aggregation and weighted average viral load of city. Raw daily viral concentrations were aggregated into weekly medians and weighted average viral load of city to reduce noise from day-to-day sampling variability.
(2) Non-Parametric correlation analysis. Spearman’s rank correlation was chosen to assess trends between wastewater viral loads and clinical cases, as it is less sensitive to outliers and non-normal distributions compared to Pearson’s correlation.
(3) Monte Carlo simulation. Uncertainty in back-calculating infection rates was addressed through 10,000 iterations, incorporating variability in viral shedding rates and population estimates (NH4-N-derived). The bootstrapped confidence intervals (P5–P95) reflect the stochastic nature of these parameters.

We acknowledge that advanced methods (e.g., autoregressive models, moving averages, or machine learning) could further smooth fluctuations. However, to avoid overfitting in this initial study, we opted for conservative, interpretable techniques. In ongoing work, we are collaborating with municipal engineers to acquire hydraulic network data and testing multivariate normalization (e.g., adjusting for pH and temperature).

Additionally, we have added the limitations in the discussion section.

“Third, the measured viral concentrations in wastewater directly reflect contributions from infected individuals, without explicit adjustments for RNA degradation or in-sewer dilution. RNA degradation may lead to an underestimation of true viral loads, particularly during warmer seasons or in areas with long hydraulic retention times.” (**Line 412-416**)

**Comments from Reviewer #2:**

1. Line 148: the equation can be removed.

**AUTHOR RESPONSE:** We are truly grateful for your meticulous focus on this detail. We have removed the equation. (**Line 147-148**)

2. Line 241: numbered is repeated.

**AUTHOR RESPONSE:** We sincerely appreciate your careful attention to this detail. We have deleted the repeated numbered. (**Line 244**)

3. In Table S2, why flow (m3) converted to Liters by multiplying 10million? Results should also be checked.

**AUTHOR RESPONSE:** We sincerely appreciate the reviewer’s careful scrutiny. The original unit in Table S2 was indeed incorrectly labeled due to an oversight in unit conversion. The flow value should have been 10,000 m³ (not m³). We have corrected this error by updating the unit from "m³" to "10,000 m³" to reflect the accurate volumetric scale. We have also rechecked all calculations and units throughout the manuscript to ensure consistency. We deeply regret this oversight and thank the reviewer for their valuable feedback, which has improved the clarity of our work. (**Table S2**).

4. Line 218 and 227 needs equation number. In addition, I recommend authors use abbreviations in equation with their descriptions below the equation.

**AUTHOR RESPONSE:** We sincerely thank the reviewer for their constructive suggestions. We have added the equation number and used abbreviations in equation with their descriptions below the equation. (**Line 201, Line 216-220, Line 226-228, Line 234**)

5. Authors revised the main title, and objective of this study, to highlight the predictive option of wastewater for COVID-19. However, in this case, using absolute assumed shedding rates (for RNA and NH4 per person: Line 209 and 222) would become critical. Considering my previous comments (e.g. No. 8 and 15) that point to some uncertainties in prediction, I highly recommend authors have additional assessment to answer the following question:

How much the prediction is reliable regarding the limitations and assumptions?

Authors are better to re-assess their prediction in two or more scenarios considering a reasonable range (for example: +10% and -10%) for their assumed shedding rates, infected person, and other variables and add their results in one or two paragraphs in discussion to at least clarify the potential range of coefficients in figure 3.

**AUTHOR RESPONSE:** We sincerely appreciate the reviewer's constructive feedback and valuable suggestions to improve our manuscript. We have carefully addressed the concerns regarding prediction reliability by conducting additional sensitivity analyses as recommended.

(1) Sensitivity analysis of assumed parameters

A recent study reported the faecal SARS-CoV-2 RNA shedding rate was 82.4%, and the mean faecal production per person was 128g per day (Li Y, Du C, Lv Z, Wang F, Zhou L, Peng Y, et al. Rapid and extensive SARS-CoV-2 Omicron variant infection wave revealed by wastewater surveillance in Shenzhen following the lifting of a strict COVID-19 strategy. Sci Total Environ. 2024 Nov 1;949:175235. doi: 10.1016/j.scitotenv.2024.175235).

As suggested, we performed scenario analyses by adjusting key parameters. First, we revised faecal SARS-CoV-2 RNA shedding rate from 100% to 82.4% in the model 2. Second, we adjusted daily faecal production per person from 211g to 128g in the model 3. Third, we adjusted faecal NH4-N excretion varied ±10% from baseline values in the model 4 and model 5. The revised results demonstrated minimal variation in predicted infection rates (-0.32 <Δ < 1.35 across scenarios), indicating remarkable robustness of our predictions to parameter uncertainties (below table 1 and figure 1).

(2) Correlation with reported cases

We conducted Spearman's rank correlation analysis between our wastewater-predicted infection rates and officially reported clinical cases. The strong correlations (Spearman’s r = 0.897-0.901, p<0.001) across all scenarios further validate the predictive reliability of our model, even when accounting for parameter uncertainties.

(3) Enhanced discussion

A new paragraph explicitly addresses: “In order to ensure the stability of the study results, we conducted sensitivity analyses. The faecal SARS-CoV-2 RNA shedding rate was chosen to be 82.4%; the daily faecal production per person was 128g, and the faecal NH4-N excretion varied ±10% from baseline values. The predicted infection rates calculated according to different parameters were similar (Table S3). Therefore, the results calculated by the model were reliable.” (**Line 423-428**)

Table 1. The results in different models.

| Models | Infected cases | | Infection rates | | Spearman's r |
| --- | --- | --- | --- | --- | --- |
|  | Min | Max | Min | Max |  |
| Model 1 | 6,517 | 486,384 | 0.01 | 3.27 | 0.898 |
| Model 2 | 6,781 | 506,095 | 0.01 | 3.41 | 0.897 |
| Model 3 | 9,204 | 686,982 | 0.02 | 4.62 | 0.899 |
| Model 4 | 6,517 | 486,384 | 0.01 | 2.95 | 0.897 |
| Model 5 | 6,517 | 486,384 | 0.01 | 3.6 | 0.901 |

Model 1: Persons Infected = $\frac{Cr*Q}{Cf\text{*211}+(Cru)*1.5}$; Population size = $\frac{CN*Q}{\text{4.2g}/person\cdot\text{day}}$;

Model 2: Persons Infected = $\frac{Cr*Q}{Cf\text{*211*82.4\%}+(Cru)*1.5}$; Population size = $\frac{CN*Q}{\text{4.2g}/person\cdot\text{day}}$;

Model 3: Persons Infected = $\frac{Cr*Q}{Cf\text{*128}+(Cru)*1.5}$; Population size = $\frac{CN*Q}{\text{4.2g}/person\cdot\text{day}}$;

Model 4: Persons Infected = $\frac{Cr*Q}{Cf\text{*211}+(Cru)*1.5}$; Population size = $\frac{CN*Q}{\text{4.2g*90\%}/person\cdot\text{day}}$;

Model 5: Persons Infected = $\frac{Cr*Q}{Cf\text{*211}+(Cru)*1.5}$; Population size =;

Predicted infection rates (%)

|  | Model 1 | Model 2 | Model 3 | Model 4 | Model 5 |
| --- | --- | --- | --- | --- | --- |
| 2023-01-18 | 0.76 | 0.792867 | 1.08 | 0.69 | 0.84 |
| 2023-01-24 | 0.46 | 0.475486 | 0.65 | 0.41 | 0.5 |
| 2023-01-28 | 0.77 | 0.802655 | 1.09 | 0.69 | 0.85 |
| 2023-01-31 | 0.39 | 0.40118 | 0.54 | 0.35 | 0.42 |
| 2023-02-03 | 0.46 | 0.47906 | 0.65 | 0.41 | 0.51 |
| 2023-02-06 | 0.31 | 0.321939 | 0.44 | 0.28 | 0.34 |
| 2023-02-09 | 0.19 | 0.193197 | 0.26 | 0.17 | 0.2 |
| 2023-02-13 | 0.25 | 0.261245 | 0.35 | 0.23 | 0.28 |
| 2023-02-16 | 0.01 | 0.0122018 | 0.02 | 0.01 | 0.01 |
| 2023-02-20 | 0.11 | 0.118412 | 0.16 | 0.1 | 0.13 |
| 2023-02-23 | 0.17 | 0.178269 | 0.24 | 0.15 | 0.19 |
| 2023-02-27 | 0.14 | 0.14904 | 0.2 | 0.13 | 0.16 |
| 2023-03-02 | 0.09 | 0.0920356 | 0.12 | 0.08 | 0.1 |
| 2023-03-06 | 0.15 | 0.150935 | 0.2 | 0.13 | 0.16 |
| 2023-03-09 | 0.19 | 0.198119 | 0.27 | 0.17 | 0.21 |
| 2023-03-13 | 0.11 | 0.118842 | 0.16 | 0.1 | 0.13 |
| 2023-03-16 | 0.19 | 0.195755 | 0.27 | 0.17 | 0.21 |
| 2023-03-20 | 0.09 | 0.094114 | 0.13 | 0.08 | 0.1 |
| 2023-03-23 | 0.15 | 0.157099 | 0.21 | 0.14 | 0.17 |
| 2023-03-27 | 0.26 | 0.266949 | 0.36 | 0.23 | 0.28 |
| 2023-03-30 | 0.11 | 0.115324 | 0.16 | 0.1 | 0.12 |
| 2023-04-03 | 0.07 | 0.072711 | 0.1 | 0.06 | 0.08 |
| 2023-04-06 | 0.1 | 0.104579 | 0.14 | 0.09 | 0.11 |
| 2023-04-10 | 0.06 | 0.0606161 | 0.08 | 0.05 | 0.06 |
| 2023-04-13 | 0.04 | 0.0432497 | 0.06 | 0.04 | 0.05 |
| 2023-04-17 | 0.05 | 0.0564025 | 0.08 | 0.05 | 0.06 |
| 2023-04-20 | 0.09 | 0.093842 | 0.13 | 0.08 | 0.1 |
| 2023-04-24 | 0.19 | 0.19351 | 0.26 | 0.17 | 0.2 |
| 2023-04-27 | 0.12 | 0.122994 | 0.17 | 0.11 | 0.13 |
| 2023-05-04 | 0.4 | 0.419024 | 0.57 | 0.36 | 0.44 |
| 2023-05-06 | 0.76 | 0.78599 | 1.07 | 0.68 | 0.83 |
| 2023-05-08 | 0.63 | 0.66053 | 0.9 | 0.57 | 0.7 |
| 2023-05-11 | 1.67 | 1.733 | 2.35 | 1.5 | 1.83 |
| 2023-05-15 | 1.16 | 1.20409 | 1.63 | 1.04 | 1.27 |
| 2023-05-18 | 3.27 | 3.40644 | 4.62 | 2.95 | 3.6 |
| 2023-05-22 | 1.34 | 1.39646 | 1.9 | 1.21 | 1.48 |
| 2023-05-25 | 1.52 | 1.58041 | 2.15 | 1.37 | 1.67 |
| 2023-05-29 | 1.34 | 1.39301 | 1.89 | 1.2 | 1.47 |
| 2023-06-01 | 0.77 | 0.801476 | 1.09 | 0.69 | 0.85 |
| 2023-06-05 | 0.57 | 0.595771 | 0.81 | 0.52 | 0.63 |
| 2023-06-08 | 0.73 | 0.755914 | 1.03 | 0.65 | 0.8 |
| 2023-06-12 | 0.34 | 0.353051 | 0.48 | 0.31 | 0.37 |
| 2023-06-15 | 0.34 | 0.353497 | 0.48 | 0.31 | 0.37 |

Fig 1. Predicted infection rates in different models.
